# Non-paraneoplastic autoimmune retinopathy: multimodal testing characteristics of 13 cases

**DOI:** 10.1186/s12348-019-0171-1

**Published:** 2019-02-26

**Authors:** Saira Khanna, Aline Martins, Zackery Oakey, Mihai Mititelu

**Affiliations:** 1 Department of Ophthalmology and Visual Sciences, University of Wisconsin, 2880 University Avenue, Madison, WI 53705 USA; 2 Department of Ophthalmology, University of São Paulo, São Paulo, Brazil

**Keywords:** Autoimmune retinopathy, Non-paraneoplastic autoimmune retinopathy, AZOOR

## Abstract

**Background:**

Non-paraneoplastic autoimmune retinopathy (npAIR) is a rare autoimmune disease that primarily affects retinal photoreceptor function and results in profound and often times permanent vision loss. Delay in diagnosis and treatment initiation may contribute to the poor visual prognosis.

**Methods:**

A retrospective chart review of all patients diagnosed with autoimmune retinopathy at the University of Wisconsin-Madison Eye Clinics between January 2012 and January 2017 was performed. Twenty eyes of 15 patients had evidence of any form of autoimmune retinopathy through a combination of symptoms, ocular findings, visual fields, optical coherence tomography, fundus autofluorescence, full-field and multifocal electroretinography, and serum anti-retinal antibodies. Clinical records were also analyzed for demographic data, systemic comorbidities, visual acuity, treatment employed, and disease progression.

**Results:**

We identified 18 eyes from 13 patients who fit the criteria for non-paraneoplastic autoimmune retinopathy. Sixty-nine percent of patients were female with a mean age of symptom onset of 56.9 ± 20.3 years. Sixty-seven percent of eyes had an associated autoimmune condition, most commonly hypothyroidism. Serum testing revealed a preponderance of antibodies against carbonic anhydrase II, while imaging revealed characteristic changes. Fundus autofluorescence most commonly showed hyperautofluorescence around the macula. The delayed diagnosis led to a larger reduction in the horizontal extent of ellipsoid zone in 1-mm perifoveal area on optical coherence tomography with resulting visual decline. There was no difference in the change of visual acuity when stratifying for patients with autoimmune conditions (*p* = 0.52) or treatment status (*p* = 0.50). None of the patients who received treatment developed contralateral eye involvement or experienced disease progression based on visual acuity or symptoms.

**Conclusion:**

Non-paraneoplastic autoimmune retinopathy has a wide and often challenging to diagnose spectrum of clinical symptoms and imaging findings. Immunosuppressive therapy can be considered empiric in the face of a suggestive presentation and can be initiated after an evaluation of clinical findings and multimodal testing, though treatment does not appear to affect regeneration of the ellipsoid zone on OCT or impact visual acuity. Treatment should be primarily used to prevent disease progression and contralateral eye involvement.

**Trial registration:**

N/A

## Background

Autoimmune retinopathy (AIR) is a rare idiosyncratic spectrum of diseases that share a common set of clinical findings, associations, and symptoms that ultimately lead to retinal degeneration. There are two subtypes of paraneoplastic AIR: cancer-associated retinopathy (CAR) and melanoma-associated retinopathy (MAR) [1]. There is also non-paraneoplastic autoimmune retinopathy (npAIR), which does not have any associated underlying neoplasm. Acute zonal outer occult retinopathy (AZOOR), first described by Gass in 1992, is considered a subtype of npAIR and can present with a trizonal pattern of degeneration (involvement of outer retina, retinal pigment epithelium, and choroid), although a variety of fundus presentations have been described [1, 2]. A commonly proposed pathogenesis of these diseases involves the production of immunoglobulins known as anti-retinal antibodies (ARAs) directed toward retinal antigens, leading to the inflammatory destruction of photoreceptors and ultimately to wide-spread degenerative changes in the retina and retinal pigment epithelium [3].

Patients present with broad and subtle symptoms that include decreased vision, nyctalopia, visual field defects, and photopsia; however, in early disease, most patients’ visual acuity is preserved [3]. Diagnostic assessment of AIR is challenging and often delayed given its rarity and variety of clinical manifestations, including an unrevealing examination in many of the early states [4].

Use of multiple imaging techniques coupled with detection of circulating serum ARAs can aid in the objectivity of diagnosis and monitoring the progression of AIR, especially when clinical examination or early testing does not isolate a specific diagnosis [5–8]. However, the presence of ARAs is not considered a stand-alone, pathognomonic finding given that they are found in unaffected individuals as well as in patients suffering from other systemic or ocular conditions [4]. Multimodal imaging, including spectral domain optical coherence tomography (SD-OCT) and fundus autofluorescence (FAF), can often times reveal non-specific changes, especially early in the disease course or if patients suffer from additional confounding retinal conditions (pattern dystrophies, age-related macular degeneration, iatrogenic retinopathies, etc.)

Although observation has historically been the most common method of approaching AIR, systemic immunosuppression, including the use of steroids, has been emerging as an important modality to attempt to manage this condition [9]. Treatment options specifically target the immune response induced by ARAs and prevent further retinal degeneration and irreversible vision loss, although their outcomes have not been formally evaluated in prospective randomized trials. It has been shown that initiation of treatment before irreversible damage of the photoreceptors may aid in the recovery of vision in AIR episodes; however, to date, no single treatment modality has been proven to be fully effective or reverse photoreceptor damage [10, 11]. Overall, there is little consensus in support of a specific therapy given the diverse and non-specific manifestations in testing and patient symptoms, as well as the small number of these cases reported in the literature.

The purpose of this paper is to report on a case series of 13 patients diagnosed with npAIR at a tertiary referral center who underwent multimodal testing and received a spectrum of treatments. The three primary objectives of this study are to understand the natural history of the disease, to identify predictors of visual outcomes, and to determine if treatment is associated with anatomic and visual acuity changes.

## Methods

A retrospective medical record review was performed to identify patients with the diagnosis of AIR, including both the paraneoplastic and non-paraneoplastic subsets seen between January 2012 and January 2017 at the University of Wisconsin Department Eye Clinics. Our study received approval from the institutional review board at the University of Wisconsin and followed the tenets of the Declaration of Helsinki.

The diagnosis was made based on the criteria for AIR proposed by Ferreyera et al. that segregate evidence into “strong,” “supportive,” or “helpful” [5]. All patients without associated malignancy were included in the npAIR group. Systemic work-up was done at the discretion of the patient’s primary care provider and included the following laboratory tests: thyroid stimulating hormone, Lyme titers, anti-nuclear antibody, anti-neutrophil cytoplasmic antibodies, rheumatoid factor, quantiferon-gold, Toxoplasma titers, erythrocyte sedimentation rate, C-reactive protein, rapid plasma reagin, and fluorescent treponemal antibody absorption. In addition, based on patient-specific medical histories and risk factors, some providers ordered imaging studies including computed tomography chest and abdomen, magnetic resonance imaging of brain and orbits, and positron emission tomography scans to exclude inflammatory and malignant conditions.

All patients underwent a full comprehensive ophthalmic examination, including best-corrected Snellen visual acuity (BCVA) converted to logMAR for statistical analysis, and slit-lamp and dilated fundus exams. FAF and color fundus photography, SD-OCT (Heidelberg Engineering, Heidelberg, Germany or Carl Zeiss Ophthalmic Systems Dublin, California, USA), automated Humphrey visual field (HVF) (Carl Zeiss Meditec Inc., Dublin, CA), and/or standardized Goldmann kinetic visual field perimetry (GVF) (Goldmann; Haag Streit, Koeniz, Switzerland) were performed when available. We also obtained multifocal electroretinograms (mfERG) and full-field electroretinograms (ffERG) (UTAS-3000; LKC Technologies, Gaithersburg, MD, USA) following the International Society for Clinical Electrophysiology of Vision (ISCEV) standard.

The blood samples of seven afflicted patients (five of which fit criteria for npAIR, while two had CAR and MAR, respectively) were collected and sent to the Ocular Immunology Laboratory (Oregon Health and Science University, Portland, OR) for detection of ARAs. Antibody testing was performed using previously described techniques that employed western blot analysis using proteins extracted from human retinas and immunohistochemistry [1].

The improvement of BCVA was judged as a gain of two or more lines on the Snellen chart. Stability was judged as being within two lines on Snellen chart, and deterioration was judged as a decline of two or more lines at follow-up. Anatomic preservation assessment of retina by SD-OCT was performed to judge EZ and external limiting membrane (ELM) integrity at the baseline and at the most recent follow-up visit. This assessment was completed only for patients that had multiple SD-OCTs performed on the same type of machine (either Heidelberg or Zeiss). Images were selected along with three high-definition horizontal line scans of the macular SD-OCT, one passing through the fovea and the others immediately above and below the fovea. The ELM and EZ were evaluated qualitatively for disruption in the 1-mm-wide area centered on the fovea, particularly looking for subfoveal preservation of outer retinal elements. The horizontal extent, in microns, for these two layers was measured within the same 1-mm-area in the foveal scan, in order to have a quantitative parameter of change from baseline to follow-up.

To determine statistical significance, a bivariate linear mixed-effects model was fitted for each of the following three exploratory variables: systemic autoimmune disease, treatment, and visual acuity (VA) outcomes based on the time from symptoms to diagnosis and from the first ophthalmology visit in the University of Wisconsin system to last follow-up (up to November 2017). A random effect of the patient was included. Satterthwaite’s approximation was used to calculate the degrees of freedom, which in turn was used to calculate *p* values.

## Results

### Demographics

We evaluated 18 eyes from 13 patients who fit the criteria for npAIR [5]. Five patients had bilateral disease. Their clinical and laboratory data are summarized in Table 1.

**Table 1:** Clinical and demographic information on all 13 patients with npAIR

Patient	Age at diagnosis	Gender	Affected eye	Latency to diagnosis	Associated systemic disease	Serum anti-retinal antibodies
1	60	F	OU	6 years	Hypothyroidism	25, 30, 46, and 68 kDA
2	84	F	OS	None	Rheumatoid arthritis	Not done
3	46	F	OS	6 years	Multiple sclerosis	22, 30, 42, 44, 62, 72, and 136 kDA
4	66	M	OU	2 years	None	Not done
5	61	F	OU	2 years	Autoimmune hepatitis	30 kDA
6	73	F	OD	8 years	Hashimoto’s thyroiditis	30, 44, and 46 kDA
7	47	M	OS	1 year	Non-viral prodrome	Not done
8	62	F	OS	1 year	Hypothyroidism	30, 33, 60, and 70 kDA
9	89	M	OU	1 year	Hypothyroidism	Not done
10	66	F	OS	None	Myasthenia gravis, Graves’ disease	Not done
11	36	F	OD	6 years	Non-viral prodrome	Not done
12	26	M	OD	None	None	Not done
13	24	F	OU	41 years	Bullous pemphigoid	Not done

The cohort consists of 69% females, with a mean age of symptom onset of 56.9 ± 20.3 (range 24–85 years) and a median duration from onset of symptoms to diagnosis of approximately 2 years (range less than 1 month to 41 years). The median duration from the first visit to last follow-up/time of data analysis was 4.71 years (range 0.75–14.1 years).

A total of eight patients had associated systemic autoimmune diseases and two patients had a viral prodrome prior to the onset of symptoms as evidenced in Table 1.

### Symptoms

Patients most commonly presented with painless and progressive subacute to chronic visual deterioration. Specifically, there were complaints of photopsia (present in 50% of the eyes), as well as dyschromatopsia and nyctalopia (present in 22% of the eyes).

On follow-up, 44% of the eyes demonstrated improvement or complete resolution of symptoms. Of note, a significant portion of this subset of eyes with improved symptoms (62.5%) received some treatment. The remaining eyes (56%) had persistent symptoms from presentation through their most recent follow-up visit; however, none of these eyes experienced worsening of their symptoms.

### Visual acuity

BCVA at presentation ranged from 20/15 to 20/400. The mean BCVA at baseline was 0.242 ± 0.395 (Snellen equivalent 20/34.92) and at follow-up was 0.178 ± 0.348 (Snellen equivalent 20/30.1) in logMAR units.

As shown in Table 2, 50% of the eyes presented with VA of 20/20 or better, and the majority of the eyes (83.3%) remained stable, while 11.1% of the eyes had improved VA, and 6% of eyes experienced worsening VA over time.

**Table 2:** Change in VA stratified by autoimmune disease and treatment status of the eyes

Visual acuity	Total number of eyes	Number of eyes with associated autoimmune disease	Number of treated eyes
Improved	2	2	1
Stable	16	10	7
Worsening	2	1	1

**Fig. 1:**
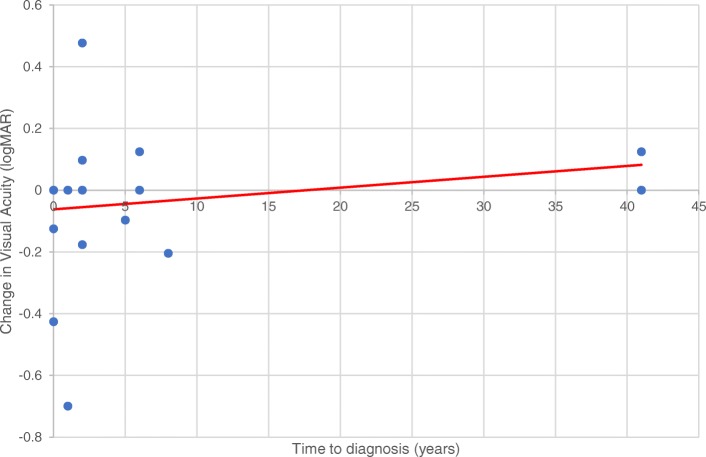
Change in visual acuity between baseline and last follow-up visit in logMAR units demonstrates a slight trend of worsening vision with delay in the time to diagnosis

There was a slight trend when analyzing visual acuity, namely, that the longer the time to diagnosis, the worse the visual outcome (Fig. 1). There was no statistical difference in baseline visual acuity between patients with autoimmune diseases and those without autoimmune conditions (*p* = 0.56). Additionally, there was no statistical difference in the change in visual acuity by autoimmune status (*p* = 0.52) or by time from initial visit to the last follow-up in the ophthalmology clinic (*p* = .92).

### Fundus photography

**Fig. 2:**
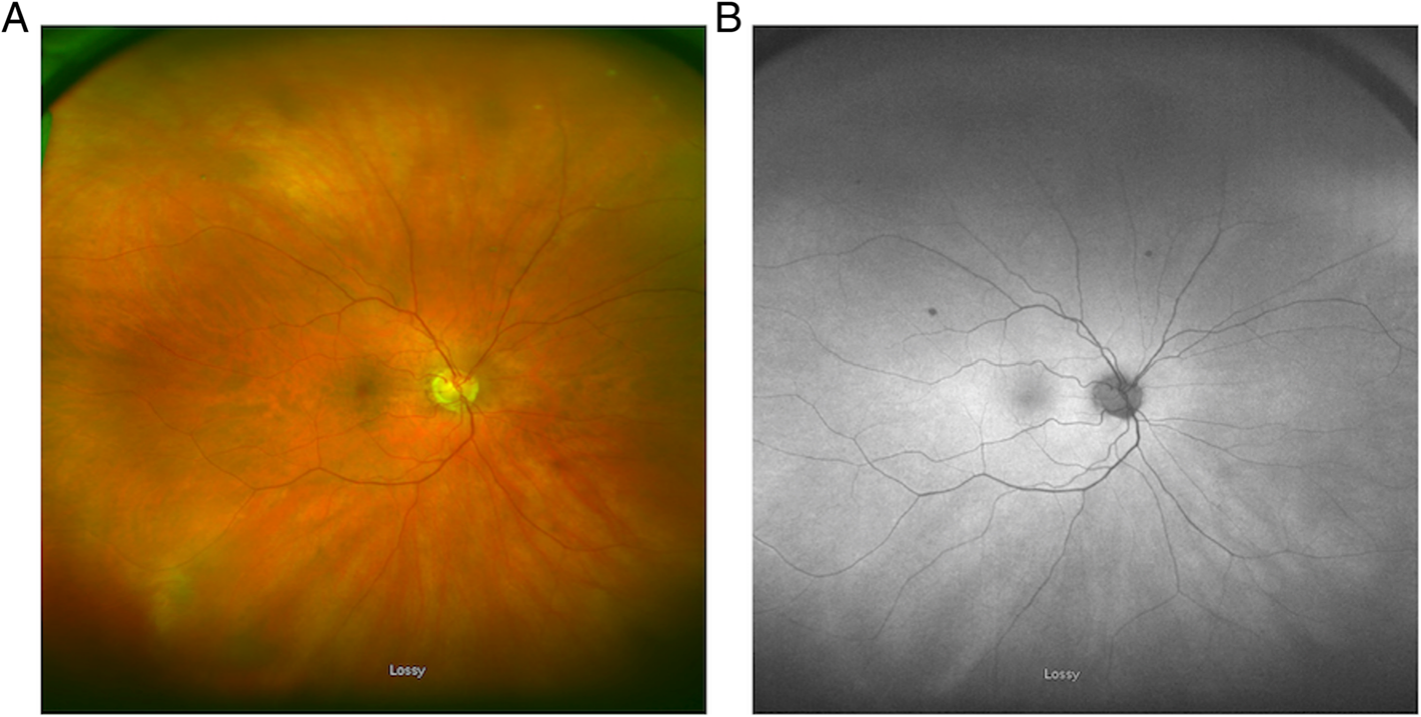
Fundus photography of patient with npAIR (patient 10). **a** Fundus photography demonstrating mild optic head nerve pallor, attenuated vasculature, and scattered RPE changes. **b** FAF demonstrated diffuse hyperautofluorescence in the macula and around the optic nerve

Fundus findings as documented by color photography ranged from normal (22% of eyes) to various retinal and pigmentary degenerative changes (Table 3). The predominant abnormal findings were pigmentary changes (61%), followed by retinal vascular attenuation (33%) and optic nerve pallor (33%). Four eyes (22%) demonstrated all these three features on the exam (Fig. 2a). There was no correlation between fundus or optic nerve changes with visual acuity or presence/type of autoimmune disease.

**Table 3:** Characteristics of fundus photography and fundus autofluorescence for all 13 patients with npAIR

Patient	Affected eye	Fundus photography	FAF
1	OU	Normal OU	Not done
2	OS	Optic nerve head pallor, attenuated vessels, RPE mottling, bone spicule-like pigment patches	Not done
3	OS	Small area of altered pigment at 2 o’clock superior and temporal to the optic nerve	Hyperautofluorescence surrounding the optic nerve and macula with normal autofluorescence inside of the ring
4	OU	Mild optic nerve head pallor, attenuated vessels, RPE mottling in mid-periphery OU	Not done
5	OU	Mild pigmentary change surrounding a few drusen in mid-periphery OU	Not done
6	OD	Mildly attenuated retinal vessels	Not done
7	OS	Normal	Normal
8	OS	Attenuated vessels, scattered RPE changes	Perivascular stippled hypoautofluorescence
9	OU	Diffuse atrophy of RPE, RPE mottling in the macula	Normal OD, peripapillary area hypoautofluorescence OS
10	OS	Mild optic nerve head pallor, attenuated vessels, scattered RPE changes	Diffuse hyperautofluorescence posterior pole extending from the macula around the arcades and optic nerve
11	OD	Normal	Hyperautofluorescence around the optic nerve and macula
12	OD	Bone spicules, rare anterior vitreous cells	Hyperautofluorescence surrounding the macula with normal autofluorescence inside the ring, speckled hypoautofluorescence superior, nasal, and inferior to the optic nerve
13	OU	Central atrophy of the RPE, optic nerve head pallor OU	Macular hypoautofluorescence OU with slight hyperautofluorescence speckled in posterior pole

### Fundus autofluorescence

**Fig. 3:**
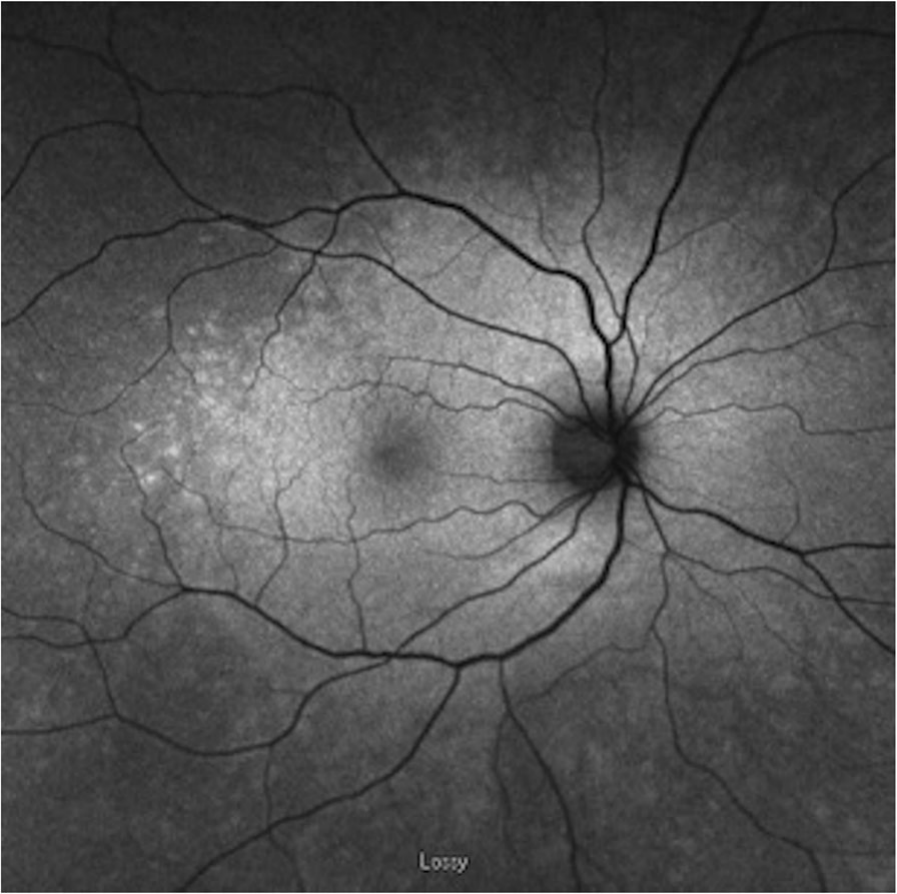
Fundus autofluorescence in patient with npAIR (patient 13). FAF demonstrates high-level detail of speckled hyperautofluorescence in the macula and around the optic nerve

More than half (56%) of the eyes underwent FAF imaging, with a range of findings (Table 3). The most common patterns included hyperautofluorescence around the macula (50%) and hypoautofluorescence around the optic nerve (30%). The FAF was unremarkable in 20% of cases. Hyperautofluorescence around the optic nerve was only found in the eyes with accompanying hyperautofluorescence of the macula (20% of eyes) (Figs. 2b and 3). Of the two eyes that were normal on FAF, one eye had improved VA and the other remained stable from baseline to follow-up.

### Visual fields

**Fig. 4:**
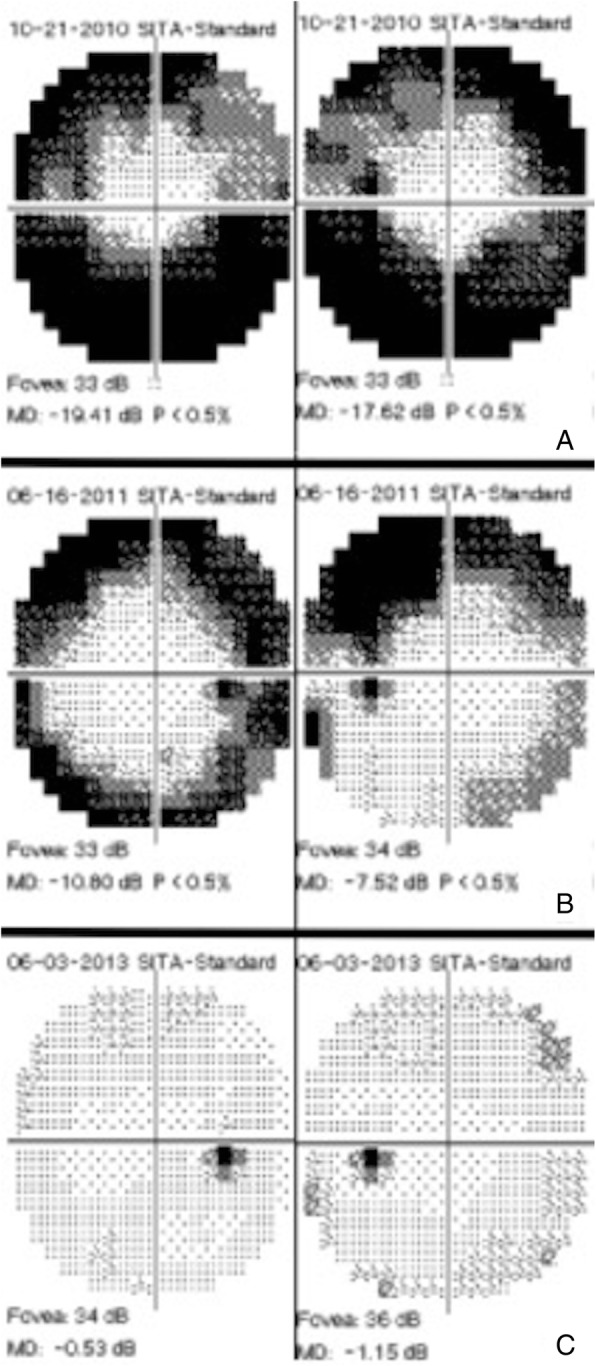
30-2 HVF of npAIR patient (patient 5) managed with valacyclovir. **a** Field defects at presentation. **b** Partial recovery of the visual fields after a 4-week course of treatment with valacyclovir. **c** Resolution of visual field defects at most recent follow-up (3 years after presentation)

At presentation, a scotoma was described in 61% of the eyes. A majority (83%) of the eyes underwent formal visual field testing, in the form of either HVF or GVF, and the most common finding was peripheral constriction (40%). VF testing was normal only in a minority of cases (7%), including the one eye of a patient without underlying autoimmune disease. An enlarged blind spot was seen in 27% eyes, while 40% demonstrated various types of scotoma (ring, superior, paracentral, central, temporal, and arcuate) on VF testing. Four eyes (two patients with bilateral disease) did show normalization of their visual fields. Autoimmune disease was a common factor in these eyes, whereas there was no correlation with treatment. Figure 4a–c demonstrates the recovery of the peripheral constriction after treatment.

### Full-field and multifocal electroretinography

A majority of the eyes underwent electrophysiologic testing. Specifically, 67% of the eyes underwent ffERG, 61% of the eyes underwent mfERG, and 50% of the eyes underwent both ffERG and mfERG. On initial ffERG testing, all eyes demonstrated amplitude reduction under scotopic and photopic conditions. Specific findings included delayed 30 Hz flicker in 16.67% of the eyes, reduction in a-wave in 17% of the eyes, and negative b-wave in 17% of the eyes. Interestingly, three eyes had normalization of their ffERGs within 2 years from presentation. These eyes also had a VA of 20/20 or better, normal mfERGs at baseline, and did not receive treatment.

**Fig. 5:**
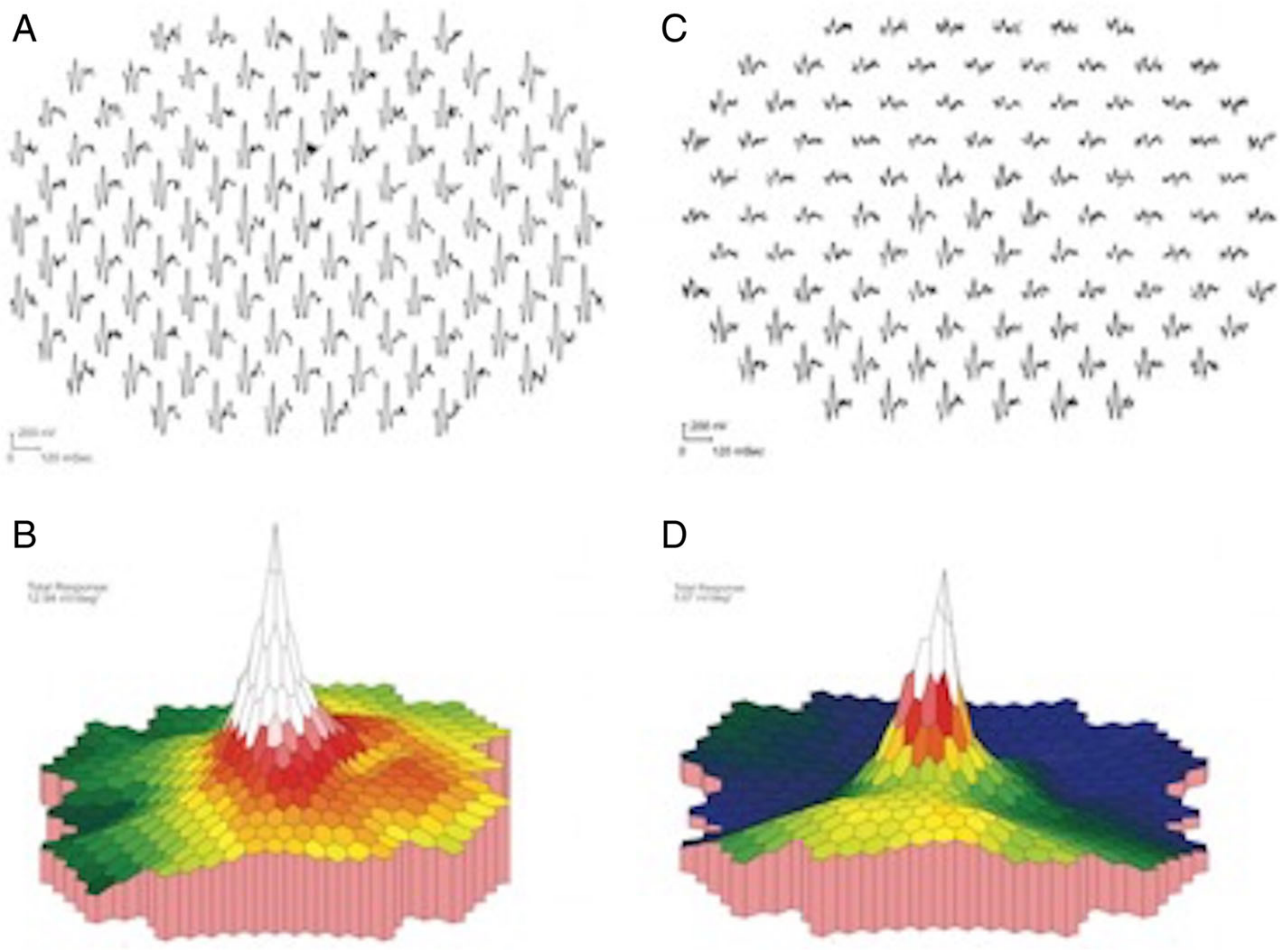
**a** Multifocal ERG traces from the right eye showing normal amplitudes throughout macula. **b** Response density surface plot of the right eye mfERG showing normal peak at fovea and symmetric amplitude decrease with increasing eccentricity from the fovea. **c** mfERG traces from the left eye showing a depression in the superior field (inferior retina). **d** Response density surface of the left eye mfERG illustrating the relatively preserved foveal response with marked loss of amplitude in the superior field (patient 3)

Testing of eyes using mfERG revealed normal findings (27%), supranormal responses centrally (18%), and diffuse amplitude reduction (46%). A representative mfERG is shown in Fig. 5a–d.

### Optical coherence tomography

SD-OCT testing was performed at least once for 88.9% of the eyes. The majority (61%) of eyes had both baseline and follow-up SD-OCT testing with the horizontal extent measurements of the EZ in the pre-defined area documented.

The most common finding on SD-OCT was attenuation of both the ONL (outer nuclear layer) and the EZ parafoveally in 56% of the eyes. Other findings on SD-OCT ranged from attenuated EZ (6%) to decreased macular thickness (11%). Only a minority of patients **(**18%) had unremarkable SD-OCT findings.

**Fig. 6:**
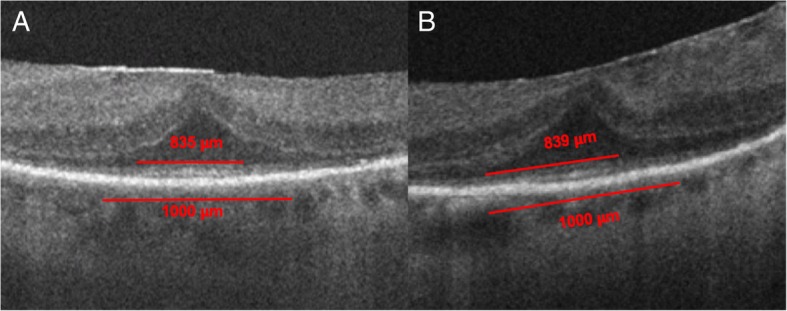
SD-OCT of patient with npAIR (patient 4) showing subfoveal preservation of outer retinal elements with loss of ONL and EZ outside the fovea. A mild, globally adherent epiretinal membrane is present without intraretinal fluid, subretinal fluid, or distortion of the outer retina. **a** Baseline measurement of EZ horizontal extent of 835 μm on SD-OCT of the right eye. **b** Follow-up measurement of the subfoveal EZ extent of 839 μm demonstrating subtle regeneration of the EZ on SD-OCT of the right eye at most recent follow-up

**Fig. 7:**
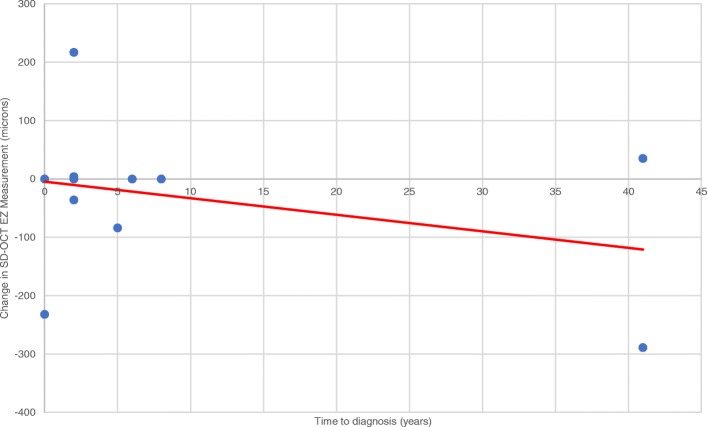
There is a slight trend of decline in SD-OCT EZ horizontal extent measurement as there is an increase in the time from onset of symptoms to formal diagnosis (*R*^2^ = .132, *p* = .271)

**Fig. 8:**
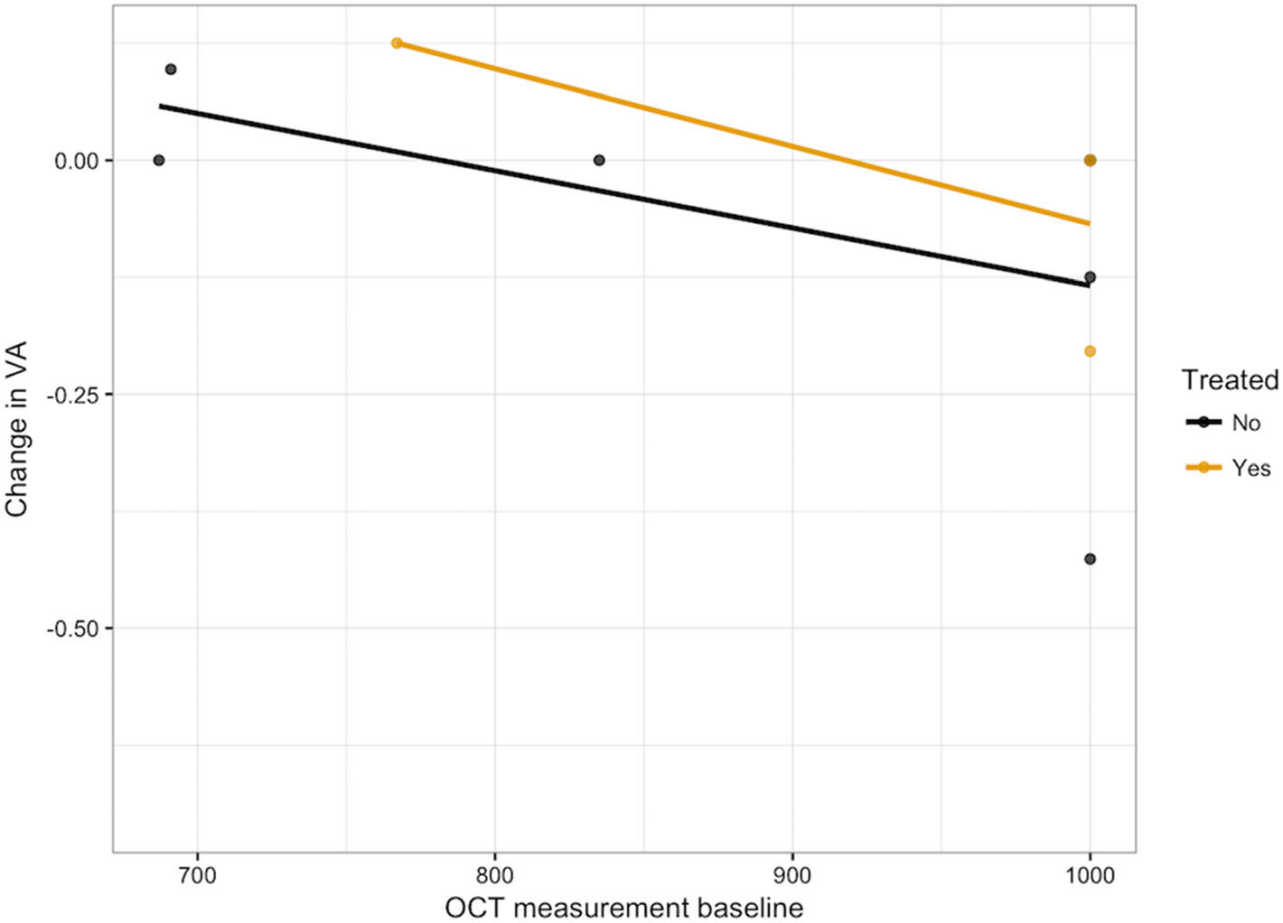
There is a subtle trend toward greater improvement in visual acuity as there is an increase in baseline measurement on SD-OCT (*R*^2^ = .50, *p* = .29 for treated; *R*^2^ = .28, *p* = .22 for untreated)

The mean horizontal extent measurement of the EZ was 907.3 ± 134.3 μm at baseline and 883.2 ± 174.4 μm at last follow-up, with a minimal average reduction of 24.1 ± 133.9 μm over time. The mean time of follow-up with SD-OCT was 1.54 ± 0.76 years. Regeneration of the EZ was seen in 23% of eyes (Table 4, Fig. 6). There was a subtle trend (*R*^2^ = .132, *p* = .271) in which a longer time to diagnosis was associated with a greater reduction of the EZ anatomic extent under the fovea (Fig. 7). We also note a correlation between a larger baseline EZ horizontal extent measurement and the magnitude of change in vision. Specifically, structural integrity and robustness of the EZ at baseline appeared to predict greater magnitude of visual acuity improvement at follow-up (Fig. 8, *R*^2^ = .50, *p* = .29 for treated; *R*^2^ = .28, *p* = .22 for untreated).

**Table 4:** Change in SD-OCT measurement by total number of eyes and number of treated eyes

EZ extent as measured on SD-OCT	Total number of eyes	Number of treated eyes
Improved	3	1
Stable	6	2
Worsened	2	1

### Anti-retinal antibodies

A total of five (38%) patients underwent serum antibody testing, while the remaining eight patients were not tested because either their symptoms had resolved or due to testing cost (Table 1).

All of the tested patients were seropositive for at least one ARA and had an average of 3.8 ± 2.2 ARAs detected. A total of 16 different antibodies were found. The most common type was the 30 kDa (carbonic anhydrase II), which was present in all of the patients who underwent testing. The other antibodies (listed in order of their prevalence) included those against 46 and 68 kDA (2 patients), and against 22, 33, 34, 35, 42, 44, 45, 60, 62, 70, 72, 80, and 136 kDA (1 patient each). All five patients had an underlying autoimmune disease and were female.

### Treatment

In our patient cohort, five patients received treatment (*n* = 7 eyes). None of the eyes without associated autoimmune disease received treatment. Three eyes from three patients received mycophenolate mofetil, with the highest daily dose of 2 g. As described in Table 5, other patients were each managed with various approaches: antioxidants (beta carotene 25,000 IU, vitamin E 400 IU, lutein 20 mg, coQ10 300 mg BID), azathioprine (titrated to 200 mg), oral prednisone (5–20 mg, intermittently), rituximab (2 doses, 1 g each), IV methylprednisone (2 boluses) followed by oral prednisolone taper and valacyclovir (3 g daily for 1 week, 1 g daily for 3 weeks).

**Table 5:** Treatment regimens for all 13 patients with npAIR with time of symptom onset to initiation of treatments

Patient	Affected eye	Treatment	Time from symptom onset to treatment
1	OU	Observation	n/a
2	OS	Observation	n/a
3	OS	Mycophenolate mofetil 500 mg BID, switched to azathioprine 200 mg	7 years
4	OU	Observation	n/a
5	OU	Valacyclovir 3 g daily 1 week, 1 g daily 21 days	2 years
6	OD	Beta carotene 25,000 IU, vitamin E 400 IU, lutein 20 mg, CoQ10 300 mg BID, mycophenolate mofetil 1000 mg BID	9 years
7	OS	Observation	n/a
8	OS	Observation	n/a
9	OU	Observation	n/a
10	OS	Mycophenolate mofetil 500 mg	6 years
11	OD	Observation	n/a
12	OD	Observation	n/a
13	OU	Rituximab (2 doses, 1 g), IV methylprednisolone (2 doses) followed by oral prednisone taper	41 years

**Fig. 9:**
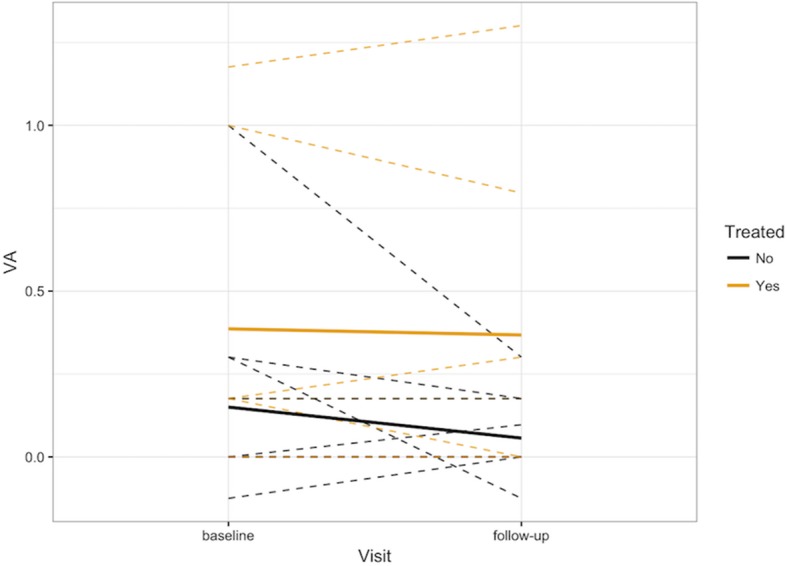
Visual acuity at baseline and follow-up visits stratified by eyes that received treatment and eyes that were observed. The baseline VA is higher for treated patients compared to those who were not treated. There is no statistically significant difference in final visual acuity of treated eyes compared to those not treated

Eyes who received treatment had worse visual acuity at baseline compared to eyes that were simply observed. Of the seven eyes that received treatment, none experienced worsening vision or contralateral eye involvement (if unilateral disease at presentation) after treatment initiation. There was no statistically significant improvement in VA between the patients who were treated and those who were not, though those treated seemed to have a smaller average magnitude of change in vision (*p* = 0.51) (Fig. 9).

Treatment did not seem to have an impact on the visual acuity outcomes when stratifying for autoimmune status and the varying lengths of EZ on SD-OCT. Furthermore, when analyzing for changes in VA or SD-OCT corresponding to specific treatments, no trend was identified. Two eyes noted subjective improvement of visual acuity immediately after receiving their respective treatments (rituximab/methylprednisolone), but neither noted any long-term improvement from treatment. Objectively, one of these two eyes demonstrated improvement on a combination of ffERG, FAF, VF, and SD-OCT.

## Discussion

In this retrospective case series of 18 eyes from 13 patients, we investigated the long-term course of the eyes with npAIR through the use of multimodal imaging and testing. The majority of the patients (69.2%) afflicted by npAIR in our cohort were women and had an average age of 56.9 years. Most cases in our series had an associated underlying autoimmune condition, most commonly hypothyroidism. Knowledge of these demographic and systemic health factors is important to the clinician and should raise suspicion for npAIR when considering the differential diagnosis of unusual cases of adult retinal degeneration and prompt systemic work-up to evaluate for underlying autoimmune, infectious, or inflammatory medical disease, including rule out of occult neoplasm.

The most common fundus findings in our cohort were related to RPE changes in the form of hyperplasia, bony spicules, or attenuation. Because the RPE is a major component of the blood-retinal barrier, it contributes to immune regulation, and it is a target of the degenerative progress [12]. As such, damage to the RPE tends to mirror the processes affecting the neurosensory retina and can serve clinically as a tool for gauging the course of this condition over time.

The same damage to the RPE was reflected in the patterns noted on autofluorescence testing. In our series, the majority of patients with abnormal FAF demonstrated a characteristic pattern of either diffuse or granular, stippled hyperautofluorescence throughout the posterior pole, primarily centered in the macula and the peripapillary region. This pattern of increased autofluorescence has been shown to be a result of metabolically hyperactive RPE due to abnormal accumulation of lipofuscin derivatives and is seen in other conditions such as hydroxychloroquine toxicity [13]. It has also been shown that hyperautofluorescence can stem from loss or thinning of the outer retinal elements, which allows for increased visibility of underlying RPE autofluorescent signal [14]. Because FAF can detect even subtle structural changes that are difficult to recognize on ophthalmoscopy, it represents an important tool in analyzing the progression of patients with AIR and should be part of the standard imaging protocol for patients with this condition.

It is worth noting that a minority of eyes (22%) in our cohort had unremarkable fundus exams, a finding which appears to be more common early in the disease course, prior to the development of irreversible retinal and pigmentary degenerative changes. This might not only make the recognition of npAIR more challenging but could also lead to delay in both diagnosis and initiation of systemic evaluation for autoimmune or neoplastic disease.

Posterior pole changes, as documented through color photography, did not show an association with vision. With only four (22%) eyes demonstrating all three major physical exam characteristics (pigmentary degeneration, vascular attenuation, and optic nerve pallor) but stable vision, our data did not isolate specific patterns of fundus changes that correlated with visual acuity.

The finding that vision is not directly associated with the severity of clinical findings reflects the multifaceted nature of the spectrum of AIR disease. Visual acuity (as a function of foveal integrity and measured by Snellen chart) can be relatively preserved despite retinal, vascular, or optic nerve changes. Nonetheless, patients may still experience visual impairment from debilitating symptoms (photopsias, nyctalopia, or dyschromatopsia) or from various types of field defects, with approximately 93% of the eyes tested showing at least one type of deficit (e.g., peripheral constriction and enlarged blind spot)

All five patients who underwent antibody testing had positive serum results. This finding provides further evidence to the claim by Qian et al. who asserted that AZOOR (whose clinical presentation is very similar to npAIR) is influenced by peripapillary leakage of ARAs into the subretinal space [15]. This also supports a more recent hypothesis, according to which the development of retinopathy is due to a largely different (hereditary) mechanism, where apoptosis of photoreceptors triggers the release of antigens and the ultimate development of ARAs. Lastly, given that all five patients had concomitant autoimmune diseases and such conditions can be associated with the development of autoantibodies, it is possible that the positive blood sample results from our patients could be secondary to cross-reactivity with systemic disease autoantibodies [16].

Although the pathogenesis of the disease process has been associated with the presence of antibodies, testing is controversial as ARAs are not always present in patients with AIR [17]. Multiple ARAs have been implicated in various forms of AIR including recoverin, alpha-enolase, arrestin, or transducin [1]. In our study of non-paraneoplastic retinopathy cases, the most common ARA type was the anti-carbonic anhydrase II (anti-30 kDa), whereas a predominance of anti-recoverin (anti-23 kDa) or anti-alpha-enolase (anti-46 kDa) antibodies would have been expected if the cohort contained primarily paraneoplastic cases. Overall, because some of these antibodies have also been identified in normal controls as well as in systemic diseases without ocular involvement, we recommend that they interpreted with respect to clinical findings and multimodal imaging and not as isolated diagnostic indicators [17, 18].

In our series, electrophysiologic testing in the form of ffERG proved to be a sensitive tool for diagnosis of npAIR, as all eyes had abnormal findings on this test at presentation, compared to 73% of eyes with abnormal results on mfERG. Although normalization of ERG has only rarely been described, in our study, there were two patients (three eyes) who demonstrated normalization of ffERG at the 2-year follow-up [19]. Neither received any treatment and one had a viral prodrome. We hypothesize that ffERG is a more sensitive test early in the disease course, as it can detect subtle and generalized decreases in retinal function before actual structural damage becomes apparent on SD-OCT or clinical exam [1]. Therefore, in addition to the already established armamentarium used for comprehensive macular evaluation (mfERG, OCT, FAF), we recommend the addition of ffERG as an important testing modality for detecting early npAIR stages or for diagnosing less clear presentations of AIR.

The patients with reported improved symptoms had received treatment (*n* = 5 eyes). There was also symptomatic improvement in the eyes that did not receive treatment in our study, a finding confirmed by other authors [20]. Interestingly, the resolution of symptoms did not necessarily correlate with improved visual acuity. For example, one eye had worsening acuity but noted improved symptoms, while neither of the two eyes that had improved visual acuity noted improvement in the symptoms, pointing to the often-times subjective and vague nature of the symptoms associated with this condition. Additionally, the two eyes from patients who had a viral prodrome (viral upper respiratory symptoms) prior to the development of npAIR had completely resolved symptoms within a month from the onset of symptoms. Although such presentations are rare, they support an infectious etiology as the basis of some select cases of npAIR. In fact, Gass et al. and Monson et al. have reported that viruses could possibly be linked to the development of AZOOR through molecular mimicry, and it appears that those patients had the quickest and most complete recovery [3, 21].

Although more than half of the patients (61%) were managed by observation only, potentially because of disease stabilization by the time they were diagnosed, the remaining 39% of patients received some form of treatment. The spectrum of treatments in our study included the use of antioxidants, immunosuppression, and even anti-virals. Although the use of systemic immunosuppression in the form of steroid-sparing agents (mycophenolate, rituximab) was the most common treatment modality, the wide spectrum of interventions used is a reflection of the complicated and challenging pathophysiologic features of AIR. While historically there has been no standardized treatment algorithm for clinical practice, more recent guidelines have generally recommended the use of corticosteroids and conventional steroid-sparing agents as first-line treatments [9].

Treatment did not appear to have a statistically significant impact on visual acuities or recovery of affected eyes in our study, since out of the eyes that received treatment, only one demonstrated objective improvement in vision as measure by logMAR. In our series, most of the patients had unilateral eye disease. We emphasize that none of the patients who presented with unilateral disease and had received treatment experienced symptom progression of disease in the other eye. Therefore, even if treatment can provide modest visual acuity improvement in already affected eyes, the decision to treat is primarily to prevent disease progression and contralateral eye involvement.

SD-OCT provides objective measures of retinal damage and may offer clues toward the diagnosis of autoimmune retinopathy. Outer retinal abnormalities and/or decreased central macular thickness on SD-OCT were seen in all patients in Abazari et al. [22]. In our study, the most common pattern of retinal damage on SD-OCT was attenuation of ONL and EZ, with relative foveal preservation of the outer retinal elements (Fig. 6). For the five treated eyes that we were able to evaluate with baseline and follow-up scans, the range of treatment durations before most recent follow-up SD-OCT was 2 months to 28 months. Treatment did not appear to be associated with an increase in the anatomic extent or robustness of the EZ. It is possible that the treatment duration of these patients was not long enough to assess for anatomic changes on SD-OCT or that their condition was either quiescent or unresponsive to the individual treatment received. Because the photoreceptor outer segments can undergo renewal, it is possible that the damage is reversible with the help of immunosuppressive therapy early on in the disease course. This may be detected structurally on SD-OCT, but it is unclear how long this process takes and if other requirements (such as removal of ARAs from circulation) need to be met for this regeneration to occur [23]. Although at time points evaluated in this study treatment did not appear to have an effect on vision or imaging, there may be long-term benefits when treatment is initiated earlier in the disease process and is maintained for longer periods of time.

Our data seemed to suggest that the longer time to diagnosis led to both visual acuity deterioration and greater reduction in the subfoveal EZ extent on SD-OCT measurements over time (Figs. 1 and 7). While the longest time to diagnosis in our cohort was seen in one patient with bilateral involvement, we believe that these specific trends would be reinforced if the study contained more eyes with a longer follow-up window and a longer time to diagnosis, which has often been reflected in the general clinical practice. Thus, in keeping with a median time to diagnosis of 2 years, this suggests that delay in diagnosis is an important challenge in the management of npAIR. It is also possible that this interval could be the amount of time at which regeneration of the photoreceptor outer segments cannot occur anymore. Additionally, in our study, the greater the size of the EZ on baseline SD-OCT appeared to be correlated with better visual acuity, which underscores the role of SD-OCT as a key imaging modality in assessing for symptomatic changes and for monitoring visual progress.

Interestingly, although on average, there was an insignificant decrease in EZ extent over the time, we noted regeneration of the EZ in the three eyes. Matsui et al. did demonstrate in AZOOR that at 6 months, the ELM and EZ can regenerate; however, they did not correlate their findings with treatment [24]. Other articles have shown recovery of the EZ and VA after intravitreal injection of dexamethasone (AZOOR) and recovery of the EZ reflectivity with difluprednate (npAIR) [25, 26]. In hydroxychloroquine toxicity, the integrity of the ELM predicts regeneration of the EZ [13]. In our study, eyes where there was regeneration of the EZ had milder forms of npAIR and had intact ELMs overlying the EZ, suggesting a common pathway to other similar maculopathies. Only one eye received treatment. This shows that in the rare cases that may experience some recovery, there are potentially other variables influencing this process, such as the time to diagnosis, choice and duration of treatment, and management of associated systemic disease.

Overall, the majority of patients in our study did not show progression on clinical exam or based on various retinal testing or imaging modalities such as ERG, FAF, or SD-OCT. It appears that after an initial decline early upon onset of npAIR, there is a long-term course of stabilization. Of note, the treated eyes tended to have worse baseline BCVA and a smaller magnitude of improvement of their vision. The poorer vision at baseline in patients receiving some form of treatment can be due to different causes, including delay in diagnosis, postponing treatment until there is visual deterioration, or simply the providers’ lack of familiarity with npAIR and the use of observation as historically the most common method of approaching this condition.

**Fig. 10:**
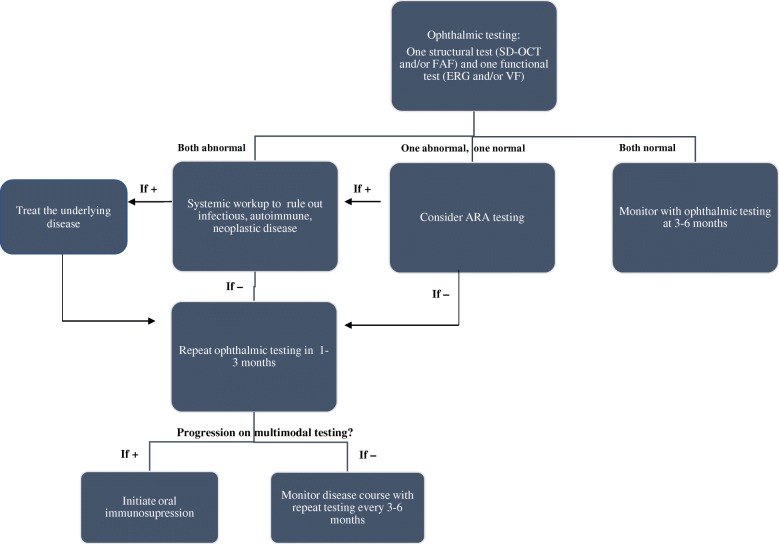
Approach to diagnosis and management for npAIR

Based on our findings from ophthalmic examination, patient symptoms, multimodal imaging, and laboratory testing, we propose a straightforward algorithm for use in clinical practice to determine diagnosis and initiation of treatment for npAIR (Fig. 10). There should be at least two abnormal, correlated test results that are suspicious of the condition. Specifically, there should be evidence of pathognomonic findings from at least one objective, structural test (SD-OCT and/or FAF) and one objective, functional test (ERG taking preference to VF, if available). Because antibody testing is cost-prohibitive, takes time, and is not always specific, it should not be used for routine screening, but should be ordered when there is clinical suspicion in the presence of equivocal multimodal testing. Clinicians should make this diagnosis in the absence of both inherited retinal disease (e.g., retinitis pigmentosa) and concurrent use of retinotoxic medications (e.g., chloroquine and hydroxychloroquine) which can confound the diagnosis and make it even more challenging. Findings should be consistently documented on two separate, consecutive visits at least 1 month apart, although in this condition, long-term follow-up to detect subtle, real trends is important. Upon diagnosis, systemic work-up should be completed first, followed by initiation of treatment, in the form of oral immunosuppression, in the presence of progression documented on consecutive visits. As shown in our npAIR cohort, treatment ensures stability of symptoms, prevents progression and involvement of the second eye in unilateral cases.

Limitations of our study include its small size (impacting the statistical significance and strength of trends observed), retrospective nature, and the lack of standardized diagnostic, laboratory, imaging, and electrophysiologic testing protocols due to the inherent differences in the practice patterns of different physicians managing these patients. Specifically, not all the patients had ARA testing completed, largely in part because of the cost of such testing. Although potentially useful, fluorescein angiograms were not routinely obtained, as other less-invasive imaging modalities were primarily used for this diagnosis. Additionally, because of the rare nature of the condition, the wide clinical spectrum of presentation and the paucity of studies, there are no standardized treatment guidelines, and our cohort reflects the diversity of treatment modalities employed by different providers when managing this challenging disease entity in real-life conditions.

A final limitation of our study is not knowing whether the duration of systemic immunosuppression was long enough to determine sustained structural and physiologic improvements as reflected by multimodal imaging. Given the rarity of the condition, we recommend larger scale, multicenter, collaborative, and prospective studies to evaluate the effects of systemic immunosuppressive regimens on results from various retinal imaging modalities and ultimately on retinal function.

## Conclusion

This study shows the complex range of clinical and imaging findings typically seen in npAIR. Multimodal testing revealed RPE changes on color fundus photography, macular and peripapillary hyperautofluorescence on FAF, peripheral constriction on VF, uniformly reduced amplitudes on ffERG, and attenuation of the ONL and EZ on SD-OCT as the most characteristic findings. Delayed diagnosis of this condition seemed to be associated with a larger reduction in the horizontal extent of EZ on SD-OCT with resulting visual decline. There were no statistically significant changes in visual acuity regardless of the presence of concurrent autoimmune disease or whether or not patients received treatment. More importantly, although the use of systemic immunosuppression did not produce changes in the appearance of the retina between initial imaging to final follow-up, treatment in all patients with npAIR ensured stability of symptoms, prevented progression and involvement of the second eye in unilateral cases. Taken together, these results show that early npAIR diagnosis using multimodal techniques may prove to be beneficial in stabilizing vision, initiating prompt treatment, and preventing contralateral eye involvement.

## Abbreviations

AIR: Autoimmune retinopathy
ARA: Anti-retinal antibodies
AZOOR: Acute zonal occult outer retinopathy
BCVA: Best-corrected visual acuity
CAR: Cancer-associated retinopathy
ELM: External limiting membrane
ERG: Electroretinography
EZ: Ellipsoid zone
FAF: Fundus autofluorescence
ffERG: Full-field electroretinography
GVF: Goldmann visual field
HVF: Humphrey visual field
MAR: Melanoma-associated retinopathy
mfERG: Multifocal electroretinography
ONL: Outer nuclear layer
RPE: Retinal pigment epithelium
SD-OCT: Spectral-domain optical coherence tomography
VA: Visual acuity
